# Differential effects of an experimental model of prolonged sleep disturbance on inflammation in healthy females and males

**DOI:** 10.1093/pnasnexus/pgac004

**Published:** 2022-03-10

**Authors:** Luciana Besedovsky, Rammy Dang, Larissa C. Engert, Michael R. Goldstein, Jaime K. Devine, Suzanne M. Bertisch, Janet M. Mullington, Norah Simpson, Monika Haack

**Affiliations:** a Harvard Medical School, Boston, MA 02115, USA; b Department of Neurology, Beth Israel Deaconess Medical Center, 330 Brookline Ave, Dana 779, Boston, MA 02215, USA; c Institute of Medical Psychology, Ludwig-Maximilians-Universität München, 80336 Munich, Germany; d Institutes for Behavior Resources, Inc., Baltimore, MD 21218, USA; e Division of Sleep and Circadian Disorders, Department of Medicine, Brigham and Women’s Hospital, Boston, MA 02115, USA; f Stanford Sleep Heath and Insomnia Program, Department of Psychiatry and Behavioral Sciences, Stanford University School of Medicine, Palo Alto, CA 94305, USA

**Keywords:** sleep disturbance, inflammation, insomnia, cytokines, sex differences

## Abstract

Sleep disturbances, including disrupted sleep and short sleep duration, are highly prevalent and are prospectively associated with an increased risk for various widespread diseases, including cardiometabolic, neurodegenerative, chronic pain, and autoimmune diseases. Systemic inflammation, which has been observed in populations experiencing sleep disturbances, may mechanistically link disturbed sleep with increased disease risks. To determine whether sleep disturbances are causally responsible for the inflammatory changes reported in population-based studies, we developed a 19-day in-hospital experimental model of prolonged sleep disturbance inducing disrupted and shortened sleep. The model included delayed sleep onset, frequent nighttime awakenings, and advanced sleep offset, interspersed with intermittent nights of undisturbed sleep. This pattern aimed at providing an ecologically highly valid experimental model of the typical sleep disturbances often reported in the general and patient populations. Unexpectedly, the experimental sleep disturbance model reduced several of the assessed proinflammatory markers, namely interleukin(IL)-6 production by monocytes and plasma levels of IL-6 and C-reactive protein (CRP), presumably due to intermittent increases in the counterinflammatory hormone cortisol. Striking sex differences were observed with females presenting a reduction in proinflammatory markers and males showing a predominantly proinflammatory response and reductions of cortisol levels. Our findings indicate that sleep disturbances causally dysregulate inflammatory pathways, with opposing effects in females and males. These results have the potential to advance our mechanistic understanding of the pronounced sexual dimorphism in the many diseases for which sleep disturbances are a risk factor.

## Introduction

Strong evidence has accumulated in recent years suggesting that sleep disturbances, e.g. difficulties falling asleep, frequent nighttime awakenings or a short sleep duration, prospectively increase the risk for multiple diseases, including cardiovascular, metabolic, infectious, autoimmune, psychiatric, and neurodegenerative diseases (1–9). However, the underlying mechanisms and causal determinants have not been elucidated yet. Systemic inflammation is increasingly recognized as a critical pathway for the development and progression of a number of chronic diseases and as a risk factor for elevated overall mortality (10, 11). Therefore, inflammatory changes following disturbed sleep may mechanistically link the association between poor sleep and health issues (12–14). Several epidemiological studies, indeed, show an association between disrupted or short sleep and inflammation, as indicated by increased levels of IL-6 and C-reactive protein (CRP) (15). In addition, experimental studies demonstrate that levels of inflammatory markers increase when sleep is shortened to 4–6 hours per night or under total sleep deprivation conditions (16–20). These findings have strong clinical relevance, as short and disrupted sleep are highly prevalent in modern society. For example, insomnia disorder, which is characterized by difficulty falling asleep or maintaining sleep associated with significant distress or impairment of daytime function, has a prevalence of up to 20% in the United States and worldwide (21, 22). The prevalence of objective short sleep duration (< 6 hours of sleep per night) has been reported in a recent review on population-based sleep cohorts to be even higher, up to 53% (23).

Most studies to date have investigated the effects of short sleep alone, rather than the effects of sleep disturbances such as observed in insomnia disorder, on immune parameters, although sleep disturbances appear to be more relevant than overall sleep duration for increasing inflammation (22). In addition, to our knowledge, all studies that have investigated the association between sleep disturbances and inflammation to date have been cross-sectional or epidemiological in nature. Such study designs are not sufficient to determine whether inflammatory changes are causally related to sleep disturbances, or rather to other factors, such as anxiety, depression, or pain, which frequently co-occur with disturbed sleep (24–27). To examine a potential causal relationship between sleep disturbances and inflammation, we developed a model of experimentally induced sleep disturbance designed to mimic sleep–wake patterns often found in insomnia disorder as well as among a wide range of patient populations for whom disturbed sleep is a common symptom or side effect (e.g. chronic pain) (28). This experimental model includes delayed sleep onset, frequent nighttime awakenings, and advanced sleep offset times, leading to a combination of disrupted and shortened sleep. It also includes repeated cycles of several nights with disturbed sleep followed by a night of undisturbed sleep, which is often observed in individuals with chronic sleep disturbances. This pattern is at least in part the result of a buildup of homeostatic sleep pressure during the prior nights of poor sleep, resulting in a night with improved sleep (29, 30). Experimentally modeling these repeating cycles allows for an evaluation of changes and possible adaptation or sensitization in inflammatory networks over time. We also included polysomnographic (PSG) measures of sleep to assess homeostatic sleep responses following repeated cycles of disturbed and recovery sleep, as such homeostatic responses in sleep depth may affect changes in inflammatory responses.

There are prominent sex differences in sleep parameters, with healthy females having overall better objective sleep and more slow-wave sleep, the deepest stage of sleep (31, 32), which is assumed to play an especially important role in immune functions (15). At the same time, females show a higher prevalence of insomnia disorder (32, 33), and they are also overrepresented in many inflammatory-related disorders for which sleep disturbances are common (34–36). Given that there are also prominent sex differences in immune function (37) as well as in immune responses associated with sleep quantity and quality (38–40), we explored potential sex differences in the inflammatory response to experimentally induced sleep disturbance.

## Results

**Fig. 1.**
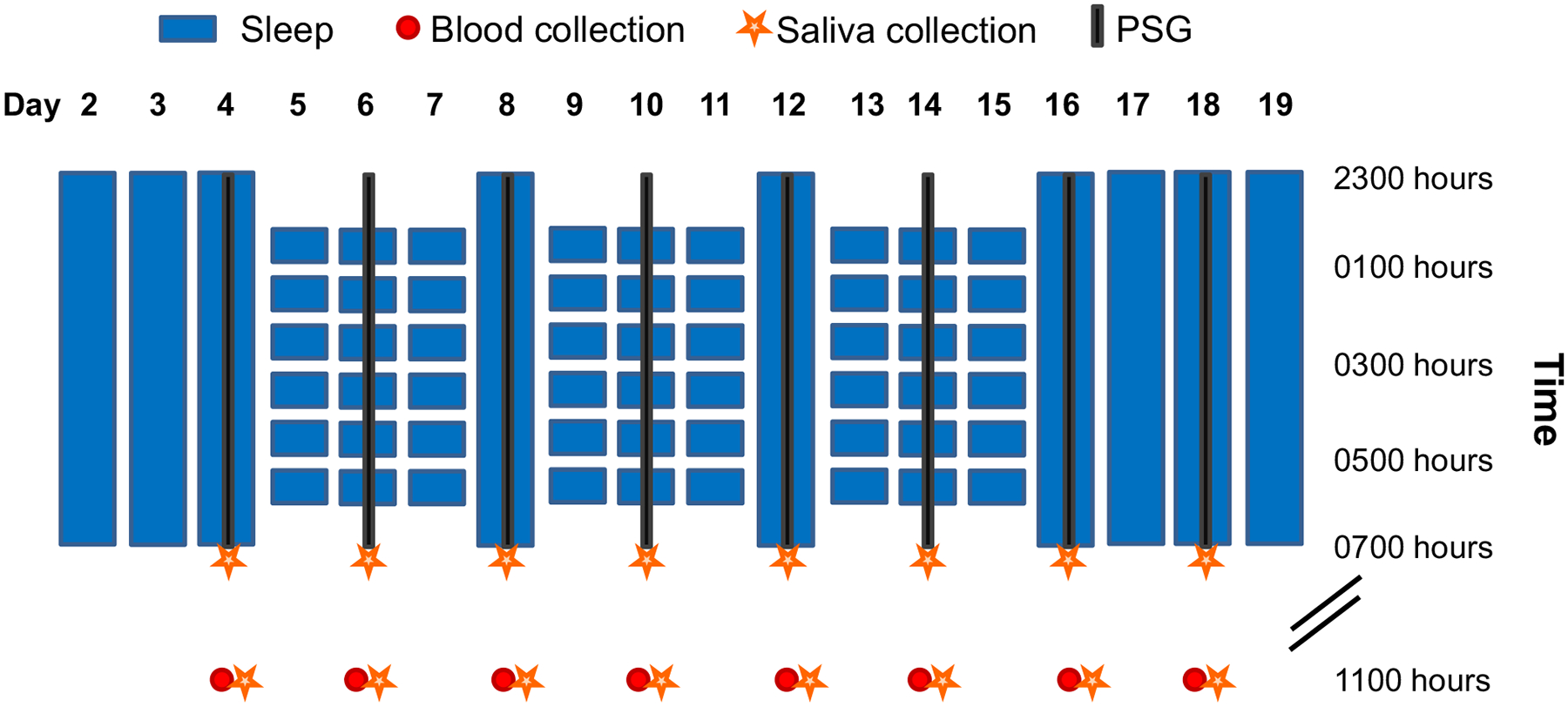
Experimental sleep disturbance (ESD) model. After 2 adaptation nights and 1 baseline night with 8-hour sleep opportunities, participants were exposed in the ESD condition to 3 sleep disturbance-recovery cycles, each consisting of 3 nights of sleep disturbance followed by 1 recovery night. The disturbance nights included a delayed sleep onset time, a disruption of the sleep period, and an advanced sleep offset, resulting in a total sleep opportunity of 4 hours. In the control condition (not shown here), participants had a sleep opportunity of 8 hours per night throughout the entire in-hospital stay. On the 8 days of intensive monitoring (days 4, 6, 8, 10, 12, 14, 16, and 18), PSG recordings were obtained at night, blood samples were collected at 1100 hours, and saliva samples were collected at 0700 and 1100 hours.

Of the 24 participants included in the analyses, 22 completed both 19-day in-hospital stays and 2 completed only 1 stay. See the Methods section for details and Fig. 1 for a graphical overview of the study protocol. Table S1 (Supplementary Material) presents baseline characteristics of the participants. Table S2 (Supplementary Material) provides an overview of *P*-values of the main and interaction effects as well as model characteristics of the generalized linear mixed model (GLMM) for all dependent variables.

### Objective measures of sleep

**Fig. 2.**
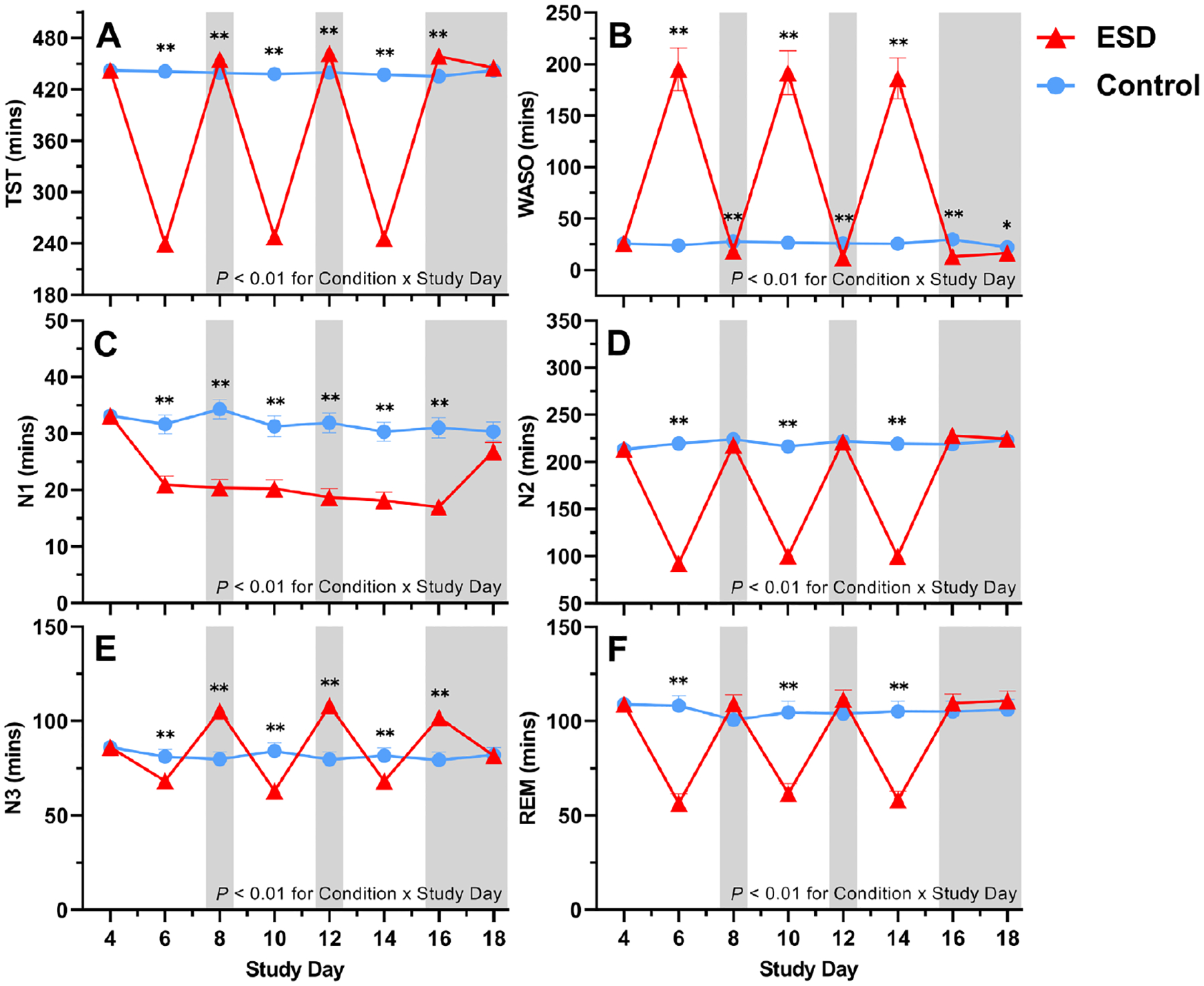
Effects of ESD on objective measures of sleep. Data present estimated marginal means ± SEM based on GLMMs for (**A**) TST, (**B**) WASO, (**C**) sleep stage N1, (**D**) sleep stage N2, (**E**) sleep stage N3, and (**F**) rapid-eye movement (REM) sleep for the ESD condition (red lines) and the control condition (blue lines). The gray shaded area indicates recovery periods with an 8-hour sleep opportunity per night. **P < 0.01, *P < 0.05, *n* = 22–24.

As expected, total sleep time (TST) was significantly reduced during the recorded sleep disturbance nights and increased during the recorded recovery nights of the experimental sleep disturbance (ESD) condition compared with the control condition (Fig. 2A). Accordingly, wake after sleep onset (WASO) was changed in the opposite direction (Fig. 2B).

Time spent in N1, N2, and REM sleep was reduced during several nights of the ESD condition (Fig. 2C, D, and F). In contrast, time spent in N3 was reduced during all disturbance nights but increased during all except the last recovery night (Fig. 2E), in line with the known strong homeostatic control of this sleep stage.

**Fig. 3.**
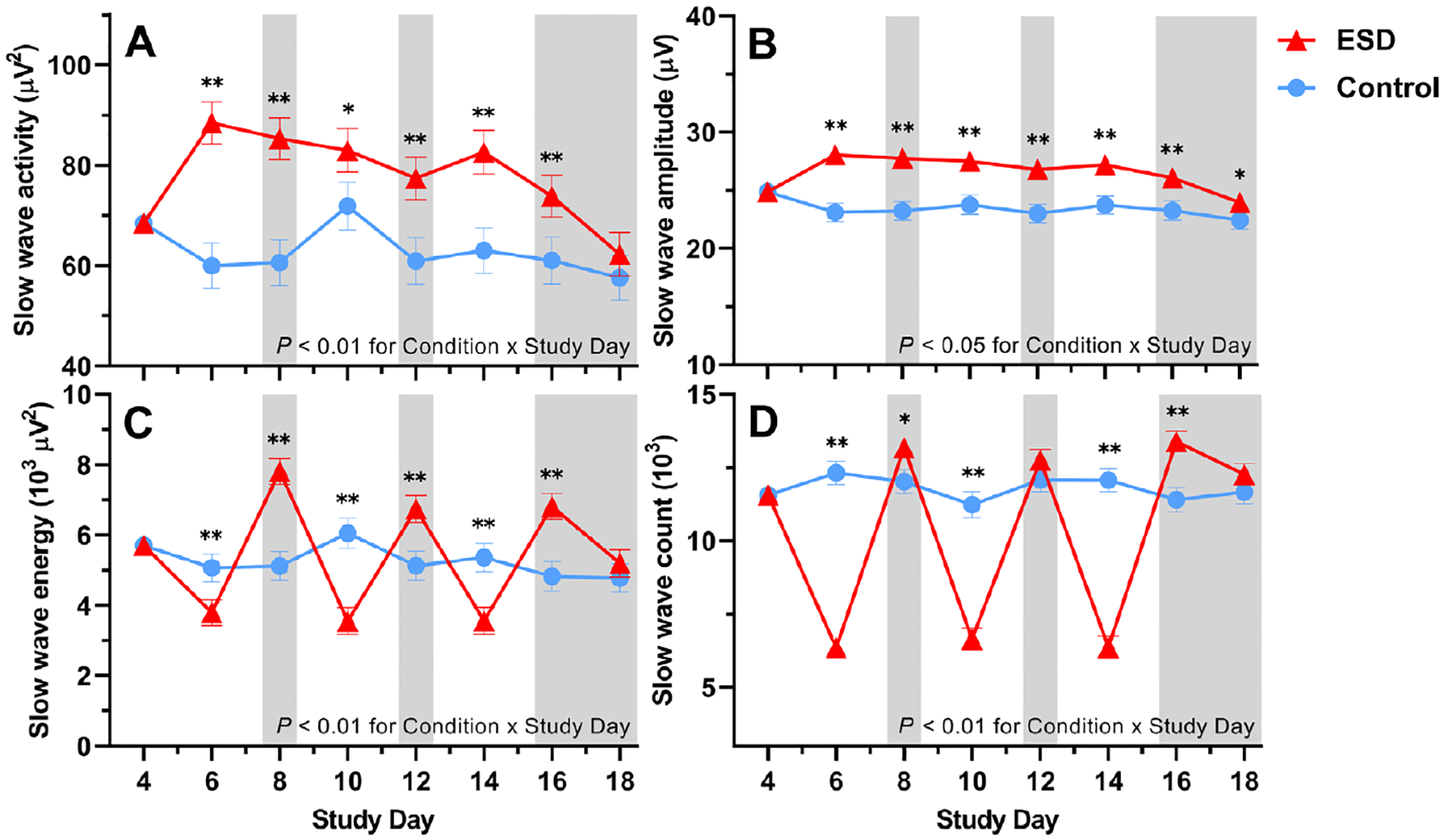
Effects of ESD on sleep depth. Data present estimated marginal means ± SEM based on GLMMs for (**A**) SWA, (**B**) slow wave amplitude, (**C**) SWE, and (**D**) slow wave count for the ESD condition (red lines) and the control condition (blue lines). The gray shaded area indicates recovery periods with an 8-hour sleep opportunity per night. **P < 0.01, *P < 0.05. *n* = 22–24.

Slow wave activity (SWA) and the amplitude of slow waves were increased throughout most or all nights of the ESD condition, reflecting deeper sleep during the available sleep opportunities of the ESD condition (Fig. 3A and B). Slow wave energy (SWE), which is a cumulative measure of sleep depth over the entire night, however, was reduced during the disturbance nights and increased during all except the last recovery night of the ESD condition, demonstrating that the ESD model was effective in reducing cumulative sleep depth during the disturbed nights despite the deeper sleep during the short available sleep opportunities (Fig. 3C). Similarly, the count of slow waves across the entire night was reduced during the disturbed nights and increased during some recovery nights of the ESD condition (Fig. 3D).

The GLMM did not indicate significant sex differences in the sleep responses to the ESD model (i.e. no significant effect for the Condition × Sex interaction), except for the time spent in N2: reductions in the duration of N2 during the sleep disturbance nights of the ESD condition were slightly less pronounced in males compared to females (Figures S1 and S2, Supplementary Material).

### Humoral immune parameters

**Fig. 4.**
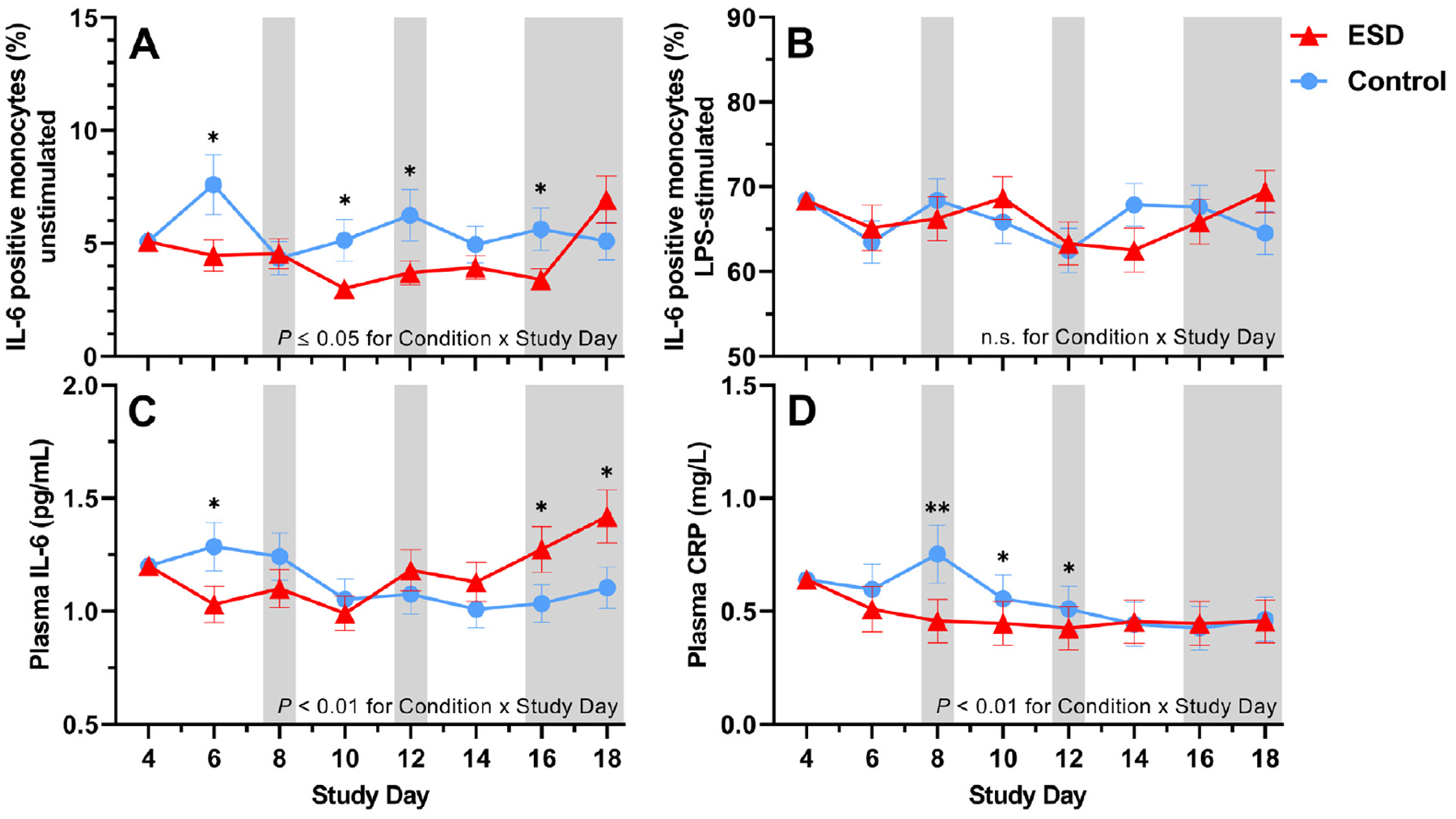
Effects of ESD on humoral immune parameters. Data present estimated marginal means ± SEM based on GLMMs for (**A**) unstimulated IL-6 positive monocytes, (**B**) LPS-stimulated IL-6 positive monocytes, (**C**) plasma IL-6 levels, and (**D**) plasma CRP levels for the ESD condition (red lines) and the control condition (blue lines). The gray shaded area indicates recovery periods with an 8-hour sleep opportunity per night; **P < 0.01, *P < 0.05; n.s., not significant. *n* = 22–24.

**Fig. 5.**
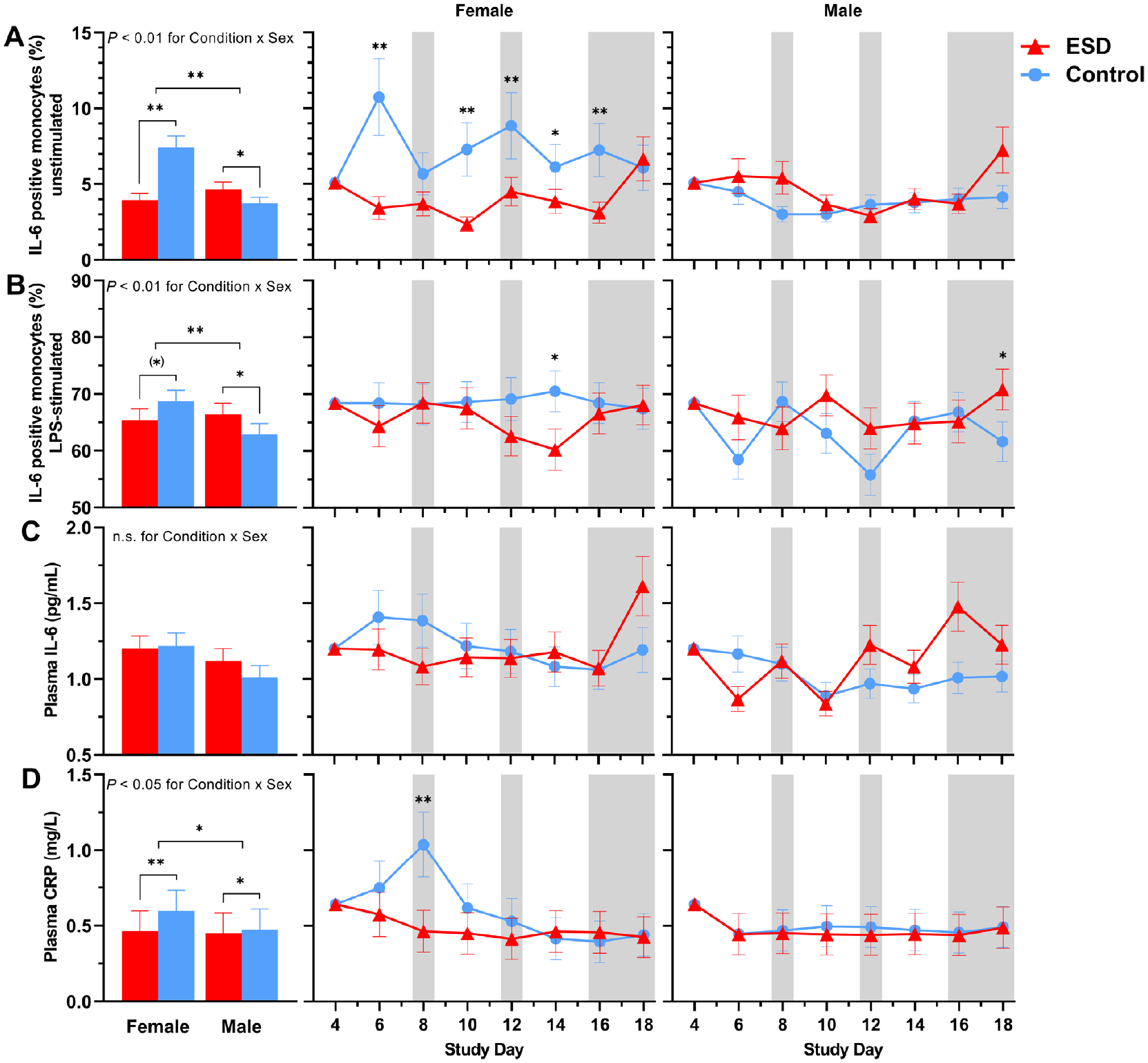
Sex differences in the effects of ESD on humoral immune parameters. Data present estimated marginal means SEM based on GLMMs for (**A**) unstimulated IL-6 positive monocytes, (**B**) LPS-stimulated IL-6 positive monocytes, (**C**) plasma IL-6 levels, and (**D**) plasma CRP levels for the ESD condition (red lines) and the control condition (blue lines) separated by sex. The gray shaded area indicates recovery periods with an 8-hour sleep opportunity per night. **P < 0.01, *P < 0.05, (*)*P <* 0.10; n.s., not significant. *n* = 22–24.

Compared with the control condition, the percentage of unstimulated IL-6-positive monocytes measured at 1100 hours was significantly reduced in the ESD condition (Fig. 4A). The reductions were observed on some sleep disturbance days but also on some recovery days. GLMM analysis of the Condition × Sex interaction revealed that the reduction in unstimulated IL-6 production during the ESD condition occurred only in females, whereas unstimulated IL-6 production was increased in males (Fig. 5A).

The percentage of lipopolysaccharide (LPS)-stimulated IL-6-positive monocytes was not affected by the ESD model (i.e. there was no significant main effect of Condition or Condition × Study Day interaction; Fig. 4B). However, there was a significant Condition × Sex interaction, wherein males had a significant increase in LPS-stimulated IL-6 production in the ESD condition, but females showed a statistical trend for a reduction of stimulated IL-6 production (Fig. 5B).

Plasma levels of IL-6 were reduced during the first sleep disturbance day but increased during the final 2 recovery days of the ESD condition compared with the control condition (Fig. 4C). The Condition × Sex interaction was not significant for plasma levels of IL-6 (Fig. 5C). Plasma levels of CRP were significantly reduced on days 8, 10, and 12 of the ESD condition (Fig. 4D). The significant Condition × Sex interaction indicated that there was a more pronounced reduction in females compared to males (Fig. 5D).

### Cellular immune parameters

**Fig. 6.**
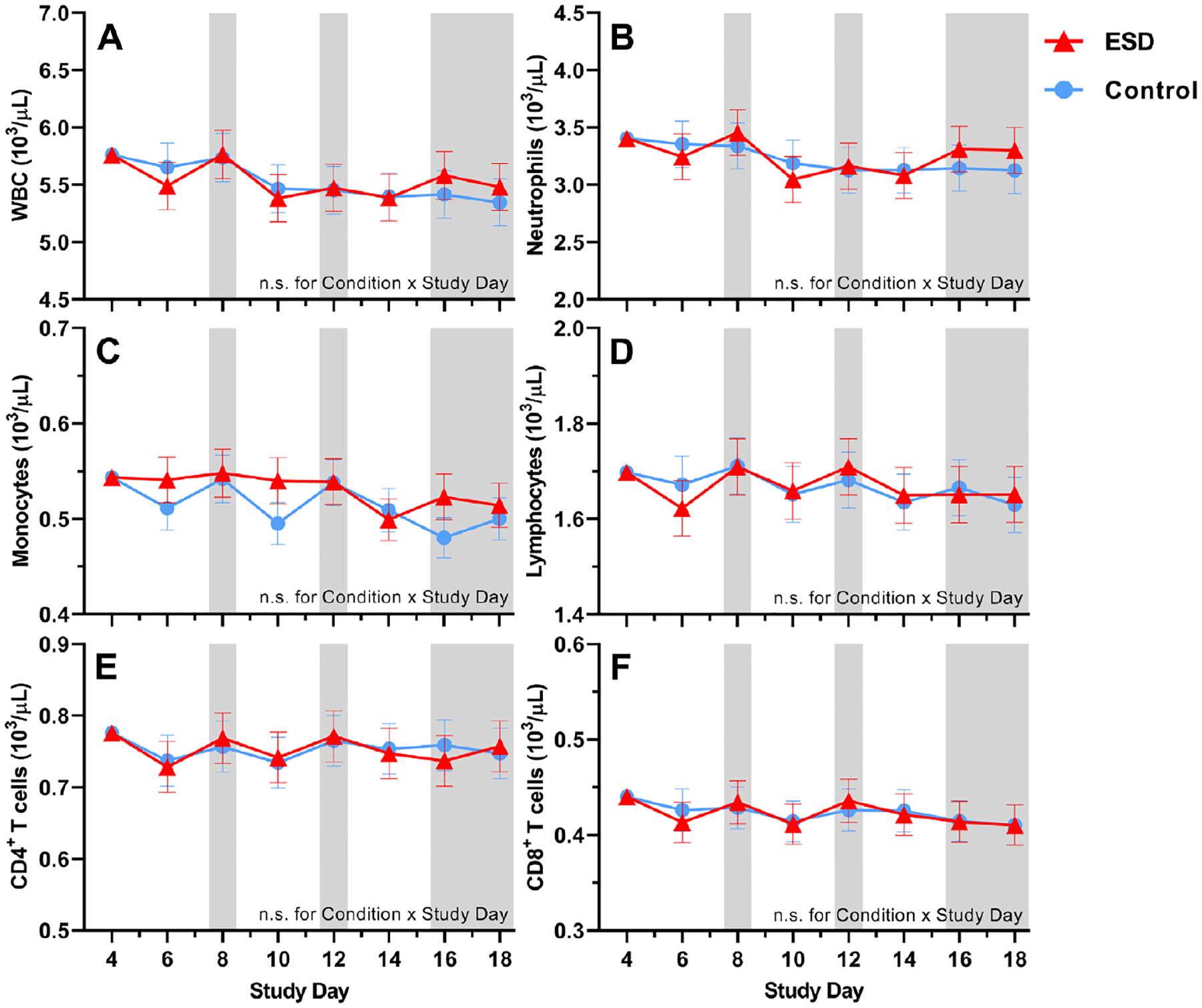
Effects of ESD on cellular immune parameters. Data present estimated marginal means ± SEM based on GLMMs for (**A**) WBCs, (**B**) neutrophils, (**C**) monocytes, (**D**) lymphocytes, (**E**) CD4^+^ T cells, and (**F**) CD8^+^ T cells for the ESD condition (red lines) and the control condition (blue lines). The gray shaded area indicates recovery periods with an 8-hour sleep opportunity per night. n.s., not significant. *n* = 22–24.

**Fig. 7.**
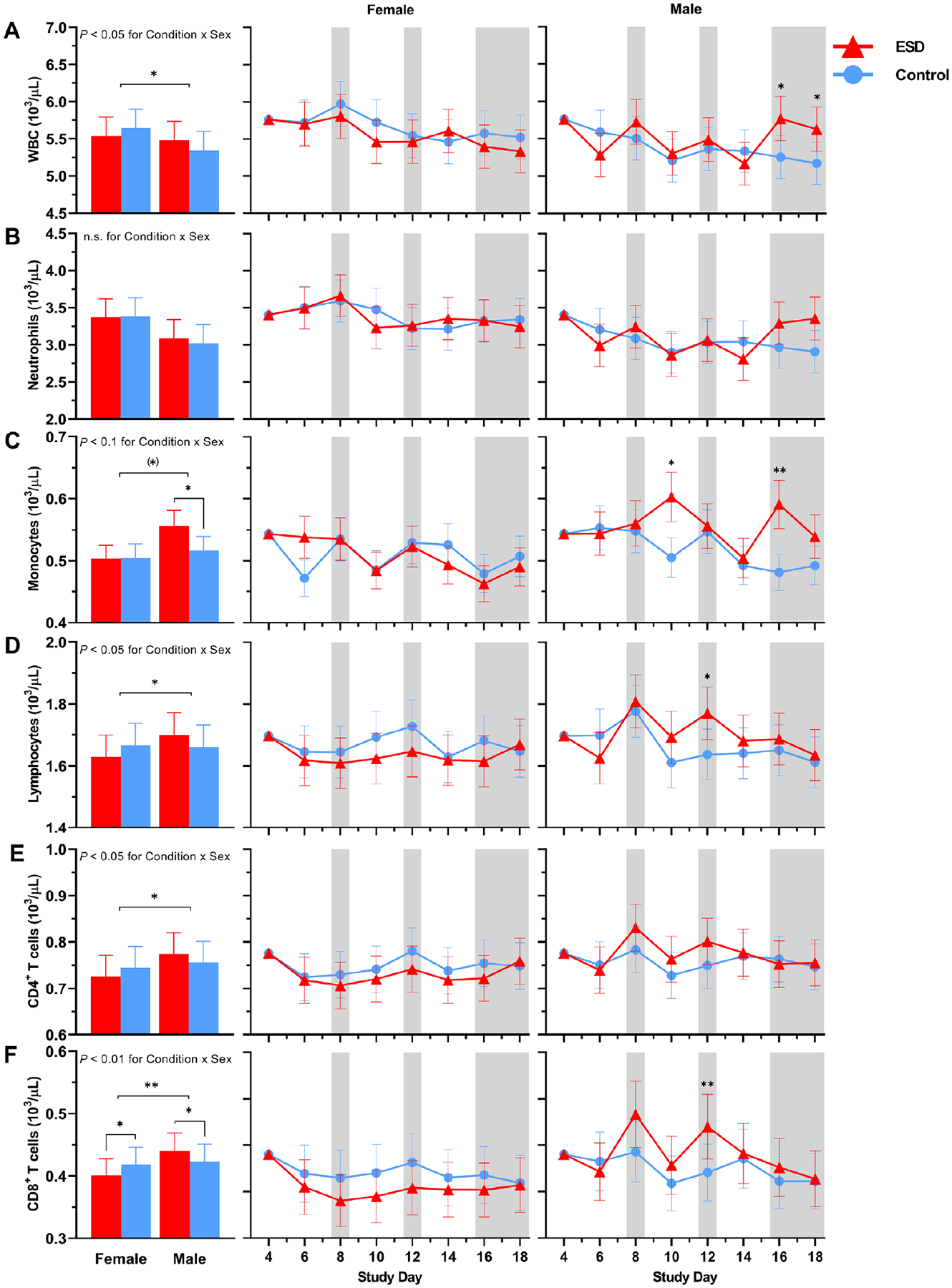
Sex differences in the effects of ESD on cellular immune parameters. Data present estimated marginal means ± SEM based on GLMMs for (**A**) WBCs, (**B**) neutrophils, (**C**) monocytes, (**D**) lymphocytes, (**E**) CD4^+^ T cells, and (**F**) CD8^+^ T cells for the ESD condition (red lines) and the control condition (blue lines) separated by sex. The gray shaded area indicates recovery periods with an 8-hour sleep opportunity per night. **P < 0.01, *P < 0.05, (*)*P* < 0.10; n.s., not significant. *n* = 22–24.

There were no significant Condition or Condition × Study Day effects for the number of total white blood cells (WBCs), neutrophils, lymphocytes, CD4^+^ T cells or CD8^+^ T cells (Fig. 6A, B, D, E, and F). However, there were significant Condition × Sex effects for all these parameters, except for neutrophils (Fig. 7A, B, D, E, and F): CD8 ^+^ T cells were increased in males but reduced in females in the ESD condition compared with the control condition. A similar pattern was observed for CD4^+^ T cells, lymphocytes, and WBCs, although the changes were not significant in the post hoc tests when looking across all experimental days.

There was a statistical trend for monocyte numbers being increased during the ESD condition (Fig. 6C). There was also a trend for the Condition × Sex interaction, with exploratory post hoc analyses indicating that monocyte numbers were significantly increased in males only (Fig. 7C).

### Cortisol levels in saliva and glucocorticoid sensitivity

**Fig. 8.**
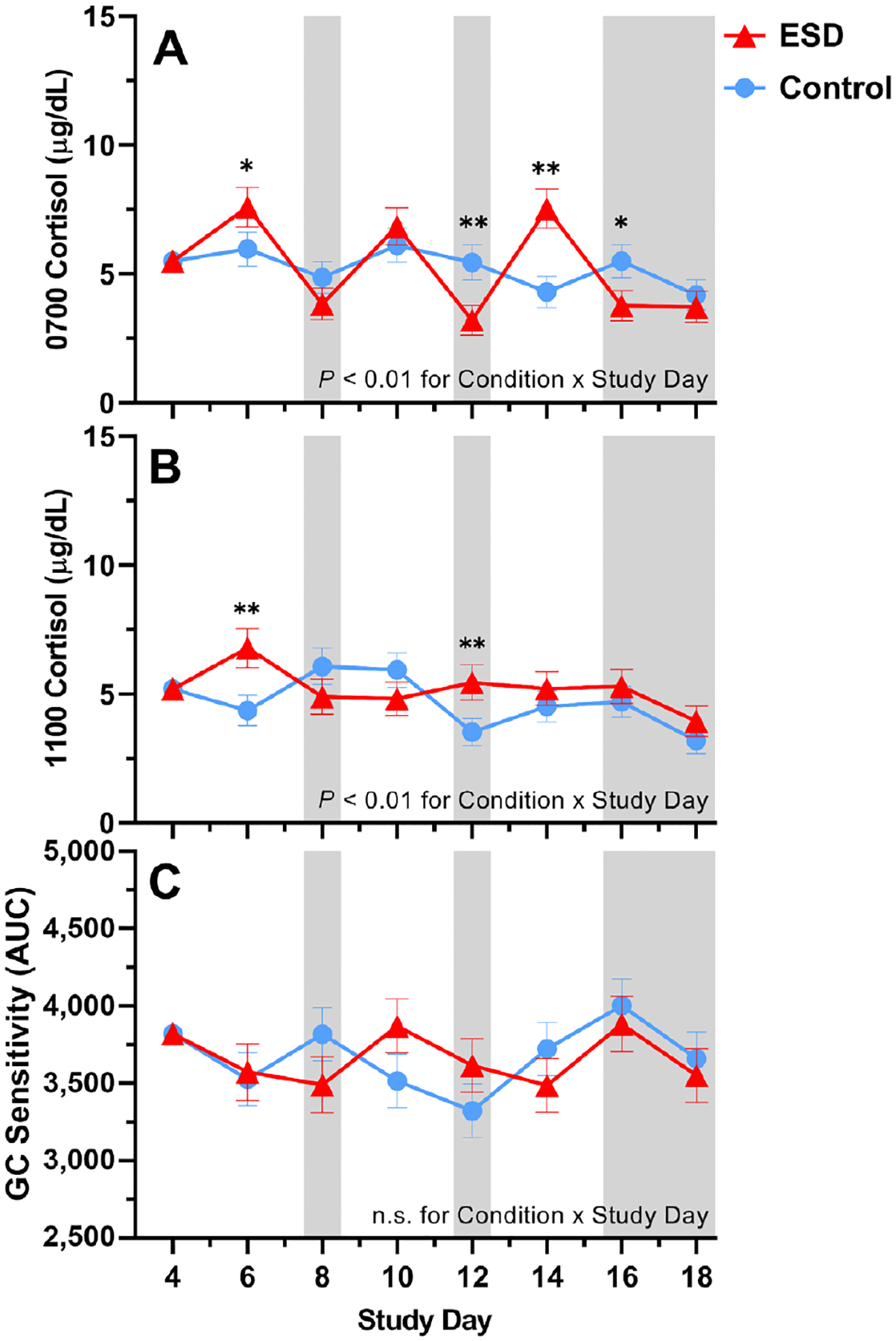
Effects of ESD on cortisol levels and GC sensitivity. Data present estimated marginal means ± SEM based on GLMMs for saliva cortisol levels at (**A**) 0700 hours and (**B**) 1100 hours, and for (**C**) GC sensitivity determined by the ability of DEX to suppress IL-6 production in monocytes for the ESD condition (red lines) and the control condition (blue lines) across all participants. Lower AUC values indicate greater GC sensitivity of monocytes. The gray shaded area indicates recovery periods with an 8-hour sleep opportunity per night. **P < 0.01, *P < 0.05; n.s., not significant. *n* = 22–24.

**Fig. 9.**
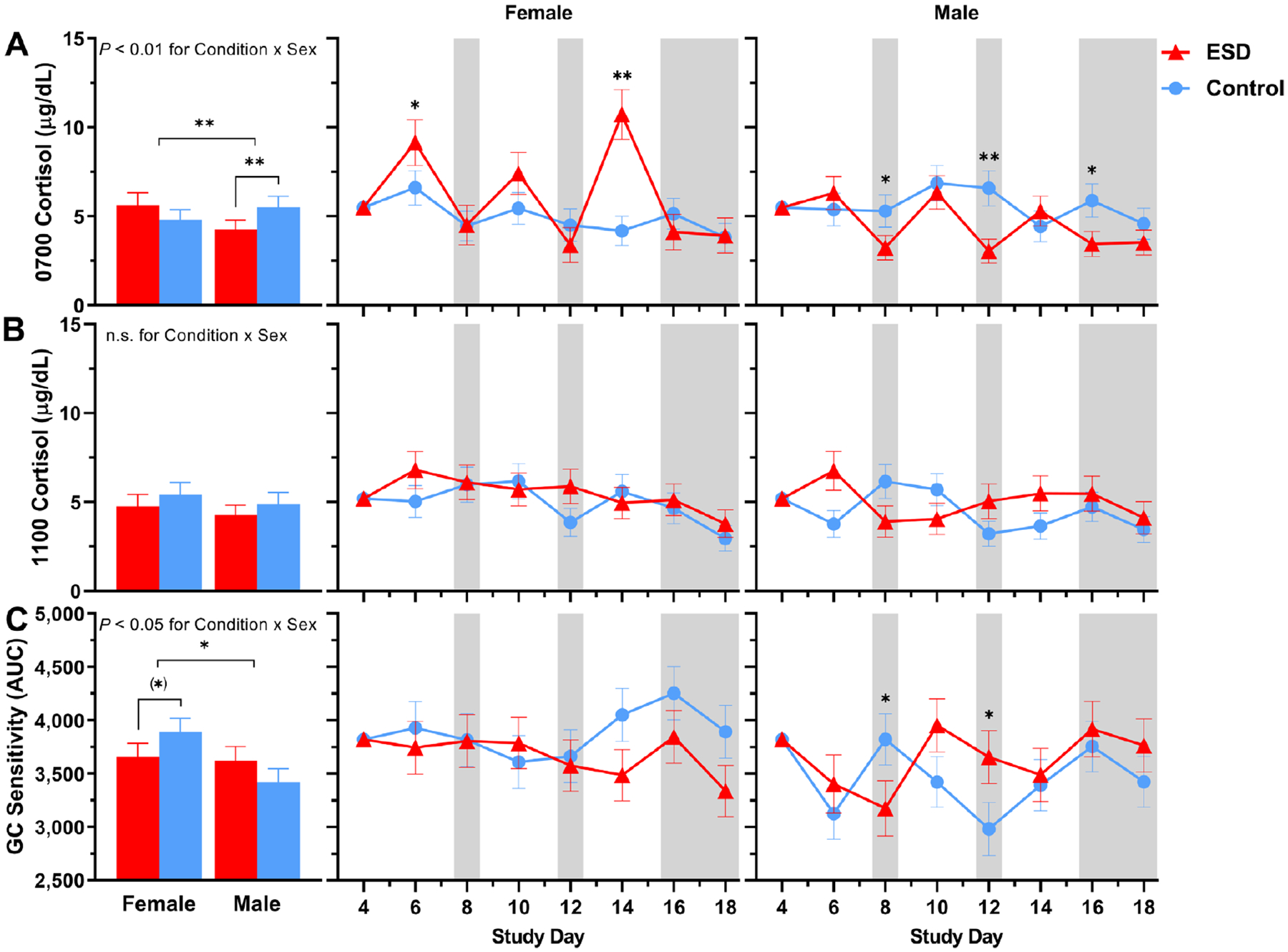
Sex differences in the effects of ESD on cortisol levels and GC sensitivity. Data present estimated marginal means ± SEM based on GLMMs for saliva cortisol levels at (**A**) 0700 hours and (**B**) 1100 hours, and for (**C**) GC sensitivity determined by the ability of DEX to suppress IL-6 production in monocytes for the ESD condition (red lines) and the control condition (blue lines) separated by sex. Lower AUC values indicate greater GC sensitivity of monocytes. The gray shaded area indicates recovery periods with an 8-hour sleep opportunity per night. **P < 0.01, *P < 0.05, (*)*P <* 0.10; n.s., not significant. *n* = 22–24.

For cortisol levels measured in fasting saliva at 0700 hours, GLMM analyses revealed significant increases on the sleep disturbance days 6 and 14 and decreases on recovery days 12 and 16 of the ESD condition (Fig. 8A). There was also a significant Condition × Sex interaction, indicating that cortisol levels were decreased in males (Fig. 9A). Exploratory analyses for single study days demonstrated that this was due to significant reductions during the recovery days of the ESD condition. In females, cortisol levels did not change significantly overall, although exploratory analyses for single study days showed significant increases on the sleep disturbance days 6 and 14. Cortisol levels measured at 1100 hours were significantly increased on the sleep disturbance day 6 and on the sleep recovery day 12 (Fig. 8B) of the ESD condition. The Condition × Sex interaction effect was not significant (Fig. 9B).

With respect to the glucocorticoid (GC) sensitivity, determined as the ability of the synthetic GC dexamethasone (DEX) to reduce IL-6 production by monocytes, no significant Condition main effect or Condition × Study Day interaction effect was found (Fig. 8C). However, there was a significant Condition × Sex effect with females showing a trend toward increased GC sensitivity in the ESD condition. The observed decrease in GC sensitivity in males was not significant (Fig. 9C).

### Subjective ratings of sleepiness, fatigue, and stress

**Fig. 10.**
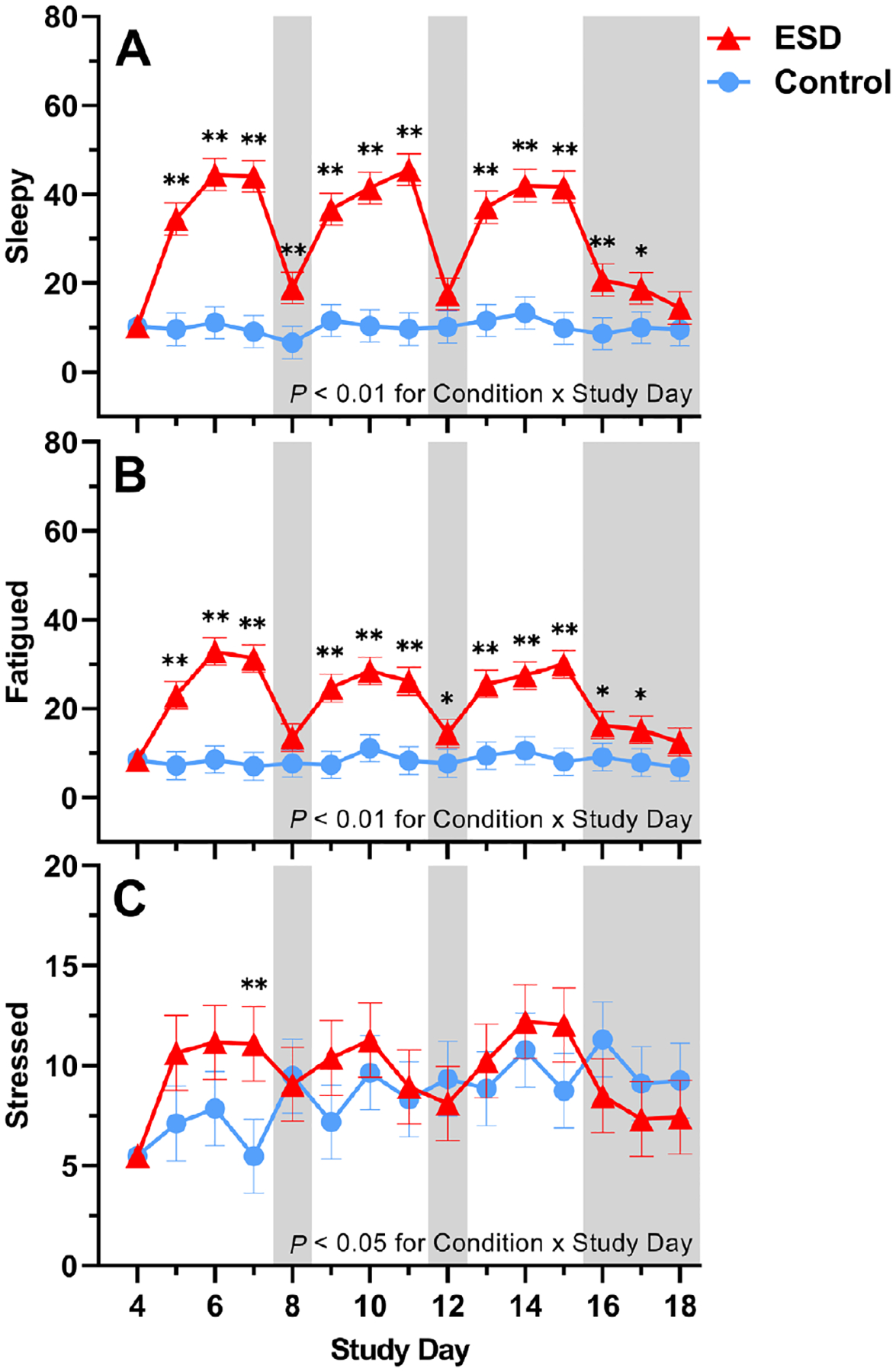
Effects of ESD on subjective feelings of sleepiness, fatigue, and stress. Data present estimated marginal means ± SEM based on GLMMs for subjective feelings of (**A**) sleepiness, (**B**) fatigue, and (**C**) stress for the ESD condition (red lines) and the control condition (blue lines). The gray shaded area indicates recovery periods with an 8-hour sleep opportunity per night. **P < 0.01, *P < 0.05. *n* = 22–24.

**Fig. 11.**
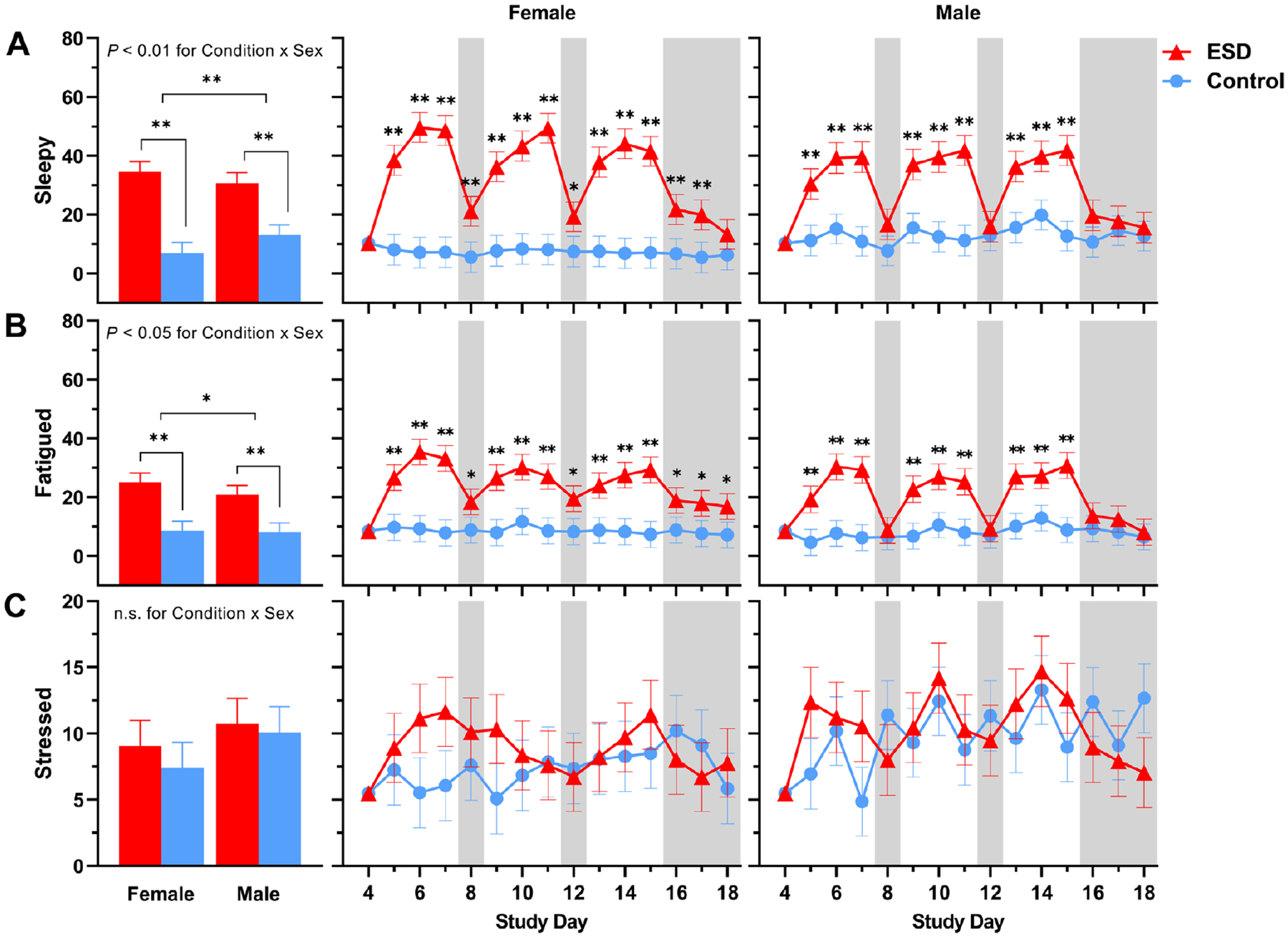
Sex differences in the effects of ESD on subjective feelings of sleepiness, fatigue, and stress. Data present estimated marginal means ± SEM based on GLMMs for subjective feelings of (**A**) sleepiness, (**B**) fatigue, and (**C**) stress for the ESD condition (red lines) and the control condition (blue lines) separated by sex. The gray shaded area indicates recovery periods with an 8-hour sleep opportunity per night. **P < 0.01, *P < 0.05; n.s., not significant. *n* = 22–24.

Subjective ratings of feeling “sleepy” and “fatigued” were significantly increased in the ESD condition compared with the control condition (Fig. 10A and B). While these subjective ratings were higher during the sleep disturbance nights than during the recovery nights, they were still elevated during some recovery nights of the ESD condition compared with the control condition. There was a significant Condition × Sex interaction effect for ratings of both sleepiness and fatigue: although ratings were increased during the ESD condition in both sexes (Fig. 11A and B), exploratory analyses for single study days indicated that, for male participants, ratings normalized after the recovery nights (i.e. there was no significant difference compared to the control condition). In female participants, ratings on most recovery nights were still significantly increased, indicating that females, in contrast to males, remained sleepy and fatigued even after 1 or more nights of recovery sleep.

Subjective ratings of feeling “stressed” were only significantly increased during the 3rd sleep disturbance night of the ESD condition (Fig. 10C), with no significant Condition × Sex interaction (Fig. 11C).

## Discussion

We tested the effects of experimentally induced sleep disturbance with intermittent recovery sleep over a 19-day in-laboratory protocol on specific immune and inflammatory markers in healthy humans. Unexpectedly, our model of ESD decreased rather than increased several inflammatory markers, including the unstimulated production of IL-6 by monocytes and plasma levels of IL-6 and CRP. Furthermore, strong sex differences in the immune responses to the sleep disturbance protocol were observed. Female participants presented an immune response pattern that could be considered as anti-inflammatory, given consistent concomitant reductions in IL-6 production by monocytes and plasma levels of CRP. In contrast, male participants presented an overall proinflammatory response, characterized by an increased stimulated as well as unstimulated IL-6 production and increases in cellular immune parameters, the most robust of which were observed for CD8^+^ T-cell and monocyte numbers.

Epidemiological studies have shown that sleep disturbances, such as those observed in individuals with insomnia disorder or those who have symptoms of insomnia, are associated with elevated inflammatory markers; however, the specific and causal role of sleep disturbance remains unknown given that sleep disturbances in insomnia disorder typically occur in the context of hyperarousal, and sleep disturbances are often comorbid with a range of diseases (15, 41). Our study provides evidence that insomnia-like sleep disturbances can cause inflammatory dys-regulation, but the findings suggest a more complex relationship than the one suggested based on findings from the epidemiological studies. In contrast to previous experimental studies exclusively manipulating sleep duration, the present study employed a model of experimentally induced sleep disturbance that exposes individuals repeatedly to both shortened and disrupted sleep. The model was designed to investigate the effects of sleep disturbances that typically occur in insomnia disorder and as a comorbidity of many diseases. Such kinds of sleep disturbances have been suggested to be more relevant for increasing inflammation than a reduction of sleep duration alone (41), and therefore, may show a stronger contribution to the development of inflammation-related diseases. Compared to models of acute sleep deprivation or restriction, models of sleep disturbance with frequent nighttime awakenings might be, overall, more stressful and invoke a larger cortisol response, which, as a consequence, can suppress inflammatory markers. Stress ratings and morning cortisol levels were indeed increased in the sleep disturbance condition. The increase in cortisol levels was due to observed increases in the female participants, who also demonstrated larger reductions in humoral inflammatory markers. However, increases in cortisol levels were restricted to 2 study days, whereas the reductions in inflammatory markers were evident across several days. This suggests that changes in cortisol were not the only factor that may explain observed changes in inflammation. Female participants also showed an increase in GC sensitivity during sleep disturbance, which could contribute to our mechanistic understanding of the exaggerated anti-inflammatory response among female participants. Alterations of hypothalamus–pituitary–adrenal (HPA) axis activity, such as manifested in elevated basal cortisol levels or cortisol responsiveness, have also been observed in insomnia patients (42, 43). Such changes are assumed to compromise health in the long term (44), and our findings suggest that females may be at a particularly high risk for adverse cortisol reactions. This assumption is in line with reports on stronger increases in the cortisol awakening response in females compared to males with insomnia disorder (45). Of note, the stronger cortisol response in the sleep disturbance condition in females was not paralleled by higher ratings of being stressed, suggesting that the biological stress response cannot be explained by the psychological stress response.

The prolonged exposure to sleep disturbances was chosen to resemble real-world sleep disturbances, which are commonly chronic in nature rather than acute. This allowed investigation of a potential habituation or sensitization of the pro- and counterinflammatory response over time during the repeated sleep disturbance challenge. Habituation of the counterinflammatory hormone cortisol has been reported in a number of studies (46), while sensitization of inflammatory mediators to a repeated psychosocial stressor has been observed in more recent research (47). However, the current study does not support a progressive increase or decrease of any mediators across the repeated exposure to sleep disturbances. This is inconsistent with our previous finding suggesting sensitization of both IL-6 and cortisol across the repeated exposure to sleep restriction over 3 weeks (20). Given that sleep duration was similarly reduced in our previous sleep restriction model and the current sleep disturbance model, sleep disturbance appears to induce qualitatively different effects on inflammatory and counterinflammatory mediators than reduction of sleep duration alone. This is a relevant finding, because it contradicts the intuitive assumption that sleep disruptions in addition to shortened sleep duration would lead to an even stronger increase in inflammation.

The current study also stresses the importance of having a within-subjects control arm with no sleep manipulation, because several immune parameters showed variability across the experimental days of the control condition. In fact, the sleep disturbance protocol appeared to reduce the variability across days observed in the control condition, which might reflect a lack of the ability of the inflammatory system to respond to changing environmental conditions, similar to reductions in heart rate variability observed in several sleep disorders (48).

The observed sex differences in the inflammatory response to sleep disturbance were strikingly large, with some of the variables showing diametrically opposed responses in females and males. Previous observational studies have also found sex differences in immune responses associated with sleep. For example, 2 studies found an association between self-reported indices of sleep disturbance and increased CRP levels in males but not in females (49, 50). Other studies report associations between subjective reports of low sleep quality and enhanced CRP or IL-6 levels only in females (40, 51–53). It is currently difficult to explain such disparate findings. However, the current findings showing that males and females mount very distinct inflammatory responses that may cancel each other out in a mixed sample can, at least in part, explain why several previous studies did in fact not find any association between sleep disturbances and inflammation (54–57).

Sex differences were also observed for ratings of sleepiness and fatigue across the repeated exposure to sleep disturbances and intermittent recovery sleep. While the magnitude of sleepiness and fatigue responses following nights of disturbed sleep was similar in females and males, there was a clear sex difference in levels of these variables following nights with undisturbed recovery sleep. Females were unable to return to their baseline values in contrast to males. Even after 2 and 3 nights of recovery sleep at the end of the protocol, females continued to report significantly elevated sleepiness and fatigue levels, respectively. This is a potentially important finding, given that sleepiness in particular has significant safety risks (e.g. risk of motor vehicle accidents) and sleepiness and fatigue both are a significant problem in many chronic diseases associated with inflammatory abnormalities, such as metabolic, neurodegenerative, psychiatric, autoimmune, and pain disorders. Many of these chronic diseases are highly comorbid with sleep disturbances (15) and are also more common in females (37). Activation of inflammatory pathways has been suggested to be responsible for the experience of fatigue, in particular fatigue associated with chronic diseases or fatigue following experimental immune activation (58). However, our current findings do not suggest that fatigue following sleep disturbances is caused by inflammatory activation. While daytime fatigue levels systematically varied as a function of disturbed and undisturbed sleep (i.e. levels increased with disturbed sleep and decreased with undisturbed recovery sleep), a similar systematic variation of inflammatory mediators could not be observed. This suggests that fatigue, at least in the context of ESDs, is not driven by peripheral inflammatory activation.

We examined the effect of our model of prolonged sleep disturbance on N3 sleep (i.e. slow-wave sleep) in more detail, because this sleep stage is assumed to be the most important sleep stage affecting the immune system (15) and negatively correlates with inflammatory cytokines (59–63). Like all other sleep stages, N3 sleep was reduced during the sleep disturbance nights of the ESD model. As expected, during the intermittent sleep recovery nights, time spent in N3 sleep as well as measures of sleep depth (i.e. SWE, SWA, slow wave count, and slow wave amplitude) were significantly increased, demonstrating the strong homeostatic control of this sleep stage. Of note, SWA and slow wave amplitude were increased also during the sleep disturbance nights, showing the ability of the participants to sleep deeper during these nights despite the frequent disruptions of their sleep. However, because of the reduced sleep opportunity, SWE and slow-wave count, both of which are cumulative measures of sleep depth, were reduced during the disturbance nights, demonstrating that the observed increase in SWA was not sufficient to fully compensate for the reduced sleep opportunity. While healthy females usually have more SWS than males (31, 32), there was no sex difference in the sleep response to the sleep disturbance model. Therefore, the observed sex effects on immune parameters seem rather unlikely to be explained by differences in the sleep response between sexes. However, this finding stands in contrast to a recent study, which demonstrated a stronger reduction in TST and N3 sleep in males compared to females following 2 nights with induced sleep disturbances (64). The small number of participants in each sex group in our study, although similar to previous sleep studies investigating sex differences in inflammatory responses (18, 39), might not have been sufficient to detect small differences in measures of sleep depth, and therefore, sex differences in these measures cannot be entirely excluded.

In conclusion, our study shows that sleep disturbances can dysregulate inflammatory pathways, however, with opposite effects in females and males. If future investigations confirm sex differential responses to sleep disturbances, with females responding with less inflammation than males, re-evaluation of the general assumption that females show stronger proinflammatory responses, which are thought to contribute to the higher prevalence of many inflammatory-related diseases in females, is needed. This assumption has already been questioned in recent reviews (65, 66), which concluded that the scarcity of sex differential data did not allow determination of whether inflammatory responses to challenges with stressors or short/disturbed sleep are stronger in females. Furthermore, the current study suggests the need to simultaneously evaluate pro- and counterinflammatory responses in future investigations of the inflammatory consequences of sleep deficiency, as this provides a more complete understanding of inflammatory dysregulation or imbalance, especially given that increases in IL-6 and CRP not always reflect an inflammatory response (67). Of note, evidence suggests that the maintenance of inflammatory balance of predominantly pro- and counterinflammatory signals is critical in disease control (68). Thus, both reduced and increased inflammatory responses to sleep disturbance can potentially affect health. Findings of this study have the potential to further our mechanistic understanding of well-known sex differences in the susceptibilities to the many inflammation-related diseases, by suggesting that the pathways through which sleep disturbances may lead to an increased disease risk differ between females and males.

The present study has some limitations. The number of participants within each sex group was low, and therefore, results on sex differences warrant replications. Although our model has a comparatively high ecological validity, real-world sleep disturbances can last for years and decades, and thus much longer than the modeled time period of 19 days. Thus, it remains unknown whether inflammatory pathways may further change beyond the experimental exposure time. Nonetheless, this study is, to our knowledge, the first to investigate inflammatory changes following more prolonged rather than acute sleep disturbances. The study was conducted in healthy and relatively young participants. While this is a limitation given the reduced generalizability to individuals with less optimal health status or of older age, the goal of this investigation was to first understand the impact of sleep disturbances themselves, in the absence of potentially interfering factors, such as comorbid disorders. The assessment of inflammatory indices was limited to plasma IL-6 levels, monocytic stimulated and unstimulated IL-6 expression, serum CRP levels, immune cell counts, salivary cortisol levels, and functional aspects of monocyte sensitivity to synthetic GCs. Rationale of focusing on these indices was that they have been well-characterized in previous models of sleep deprivation or restriction (20, 41). Future investigation will need to assess other important indices involved in inflammatory responses, to better understand the inflammatory consequences of sleep disturbances.

Taken together, findings of this study provide first evidence that prolonged sleep disturbances cause dysregulations of inflammatory pathways, with sex as a potentially strong modulator of these effects. Sleep disturbances are increasingly common worldwide and are robustly associated with an increased risk for various inflammatory-related disorders. Given that it is unlikely that we can eradicate sleep disturbances, current findings emphasize the need to identify and target inflammatory dysregulation induced by sleep disturbances, in order to limit their potential impact on disease risk and progression.

## Methods

### Participants

Participants were recruited via community and website advertisements. A total of 24 healthy young females and males were studied. A total of 22 participants completed both 19-day in-hospital stays; 2 participants completed only 1 of the 2 in-hospital conditions, 1 due to change in work/family-related requirements and 1 due to an inability to follow study procedures. See Supplementary Methods for inclusion and exclusion criteria.

### Study protocol

#### Model of ESD

This study employed a randomized, cross-over in-hospital protocol, in which each participant completed two 19-day stays: 1 sleep disturbance protocol and 1 control sleep protocol. The first 2 nights of each 19-day in-hospital stay were adaptation nights, and the 3rd night served as the baseline night, with a sleep opportunity of 8 hours (2300–0700 hours) on all 3 nights. In the **ESD condition**, insomnia-like sleep patterns were induced during the next 3 nights, with a total sleep opportunity of 4 hours/night, followed by 1 night of recovery sleep with an 8-hour undisturbed sleep opportunity. This cycle of 3 nights of disturbed sleep followed by 1 night of recovery sleep was repeated 3 times. After the 3rd sleep disturbance–recovery cycle, participants had 3 more recovery nights (Fig. 1). In the **control sleep condition**, participants received an 8-hour sleep opportunity on all nights of the protocol. The order of conditions was balanced between participants.

During each of the 9 ESD nights, the following common insomnia-like sleep patterns were induced: (a) difficulty initiating sleep, modeled with a delay in sleep onset by 1 hour (from 2300 to 0000 hours); (b) difficulty maintaining sleep, modeled with disruption of the sleep period (interval between 0000 and 0600 hours) by hourly 20 minute-awakenings, totaling 6 nighttime awakenings (5 + 1 final awakening) for a total of 2 hours of induced WASO; and (c) early morning awakenings, modeled with advancement of sleep offset by 1 hour (from 0700 to 0600 hours). The total sleep opportunity per sleep disturbance night was, thus, 4 hours. To implement nighttime awakenings, the research nurse entered the room, turned on the light to less than 20 lux, and woke up the participant by calling her/his name. During the 20-minute awakenings at night, a saliva sample was collected within 5 minutes of awakening, participants were asked to rate their well-being on computerized visual analog scales, and interacted with the attending research assistant while staying in bed in a semi-recumbent position until the next sleep opportunity began.

The 2 in-hospital conditions were separated by an interval of at least 2 months in order to allow recovery from blood sampling and from potential residual effects related to the exposure to ESD. During the two 19-day in-hospital stays, participants had 8 days of intensive monitoring (on the baseline night, every 2nd sleep disturbance day and recovery day of each of the 3 cycles, and on the 3rd recovery day at the end of the protocol, see Fig. 1). Measurements during the intensive monitoring days included PSG recordings, blood and saliva sampling, blood pressure assessment, and well-being assessment. Throughout both in-hospital stays, participants stayed in a private room at the Clinical Research Center at BIDMC. See Supplementary Methods for details on the research environment.

#### Specimen collection

Blood and saliva samplings were performed on the 8 intensive monitoring days. Participants refrained from food and fluid in-take for 60 minutes, and remained in a seated position for 15 minutes, prior to specimen collection. Blood was taken through direct venipuncture. Extracted plasma was stored at −80°C and whole blood was immediately processed for cell stimulation. Saliva was collected shortly prior to blood sampling by having participants spit in a collection tube; collected samples were then stored at −80°C.

### Measurements

#### IL-6

Stimulated and unstimulated IL-6 production by monocytes and plasma levels of IL-6 were measured at 1100 hours on the 8 intensive monitoring days using the following methods: (a) IL-6 production of monocytes (in vitro measurement): whole blood was stimulated with LPS from *Escherichia coli* O127:B8 (LPS 100 pg/mL, Sigma-Aldrich) or left unstimulated, and then brefeldin A (10 μg/mL, Sigma-Aldrich) was added to the sample, which was incubated for 4 hours at 37°C in a 5% CO_2_ atmosphere. Following fixation and permeabilization procedures (IntraPrep Permeabilization Reagent [Beckman Coulter]), fluorescence-conjugated antibodies were added (CD14 APC, CD45 KrO [both Beckman Coulter], IL-6 PE [BD Biosciences]) and samples incubated for 15 minutes at room temperature in the dark. Samples were vortexed, washed with phosphate-buffered saline solution (PBS 1X, Sigma-Aldrich), and stored at 2–8°C in the dark after re-suspension in 500 μL of PBS containing 0.5% formaldehyde. Preparations were analyzed within 24 hours using flow cytometry (Gallios, Beckman Coulter, Flow Cytometry Core at BIDMC) in 100,000 acquired events per sample. Percentage of IL-6-positive monocytes (LPS-stimulated and unstimulated) was quantified using Kaluza flow analysis software (Beckman Coulter); (b) IL-6 levels in plasma (in vivo measurement): IL-6 levels were determined by ELISA (Quantikine® HS, R&D Systems, Minneapolis, MN).

#### CRP

Plasma levels of CRP were measured at 1100 hours on the 8 intensive monitoring days through LabCorp (labcorp.com) using a high sensitivity immunochemiluminometric assay (ICMA).

#### GC sensitivity of monocytes

GC sensitivity of monocytes was determined by the capacity of the synthetic GC DEX to suppress IL-6 expression in monocytes at 1100 hours assessed on each of the 8 intensive monitoring days. Whole blood was stimulated with LPS (see above), and then different concentrations of DEX (12.5, 25, 50,100, and 200 nM; Sigma-Aldrich) as well as brefeldin A were added to the samples, which then underwent the same procedures as described above. For statistical purposes, IL-6 suppression curves were calculated by first subtracting monocytic IL-6 expression at different DEX concentrations from IL-6 expression without DEX, followed by computation of the area under the curve (AUC) using the Riemann approximation. Lower AUC values indicate greater GC sensitivity of monocytes.

#### Cortisol

Cortisol was measured in saliva using the Parameter Cortisol Assay (R&D Systems, Minneapolis, MN), and the 0700 hours (fasted) and 1100 hours sampling time of each of the 8 intensive recording days were used for analysis.

#### Blood count and blood chemistry

Numbers of WBCs including differential count, CD4^+^ T cells, and CD8^+^ T cells were analyzed in the 1100 hours blood collection through LabCorp (labcorp.com).

#### PSG recordings and slow wave analyses

PSG recordings were performed on the 8 intensive monitoring days, using Embla system N7000 (Natus Medical Inc., Pleasonton, CA). The PSG montage followed standard criteria and sleep stages were manually scored on a 30-second epoch basis (American Academy of Sleep Medicine, 2007). All PSG recordings were scored by the same sleep technician.

For slow wave analyses, channels F3 and F4 with contralateral mastoid referencing were extracted to calculate electroencephalographic (EEG) variables relevant to slow wave characteristics using Matlab version 2019b (The MathWorks, Inc., Natick, MA). EEG signals were sampled at 500 Hz and subsequently filtered using a 0.5–105 Hz bandpass and 55–65 Hz notch. Data were segmented into sequential 6-second epochs to calculate spectral power values via Fast Fourier Transformation (Welch’s averaged modified periodogram with Hamming window) and artifact removal was conducted by visual inspecting of low and high frequency power (1–4 Hz and 20–40 Hz, respectively) across all epochs of N2 and N3 sleep for each channel of a given recording and removing spikes showing a clear deviation from surrounding epochs in the time course (69). On average, 91.45 ± 9.17% of non-REM epochs were retained for analysis. SWA was defined as the 1–4 Hz delta range. Following methods utilized in prior studies (70, 71), SWE (the cumulative sum of SWA across time) was also calculated to better account for variations in sleep duration that skew SWA metrics. Slow wave morphology including count and amplitude of slow waves was examined using previously described procedures (70, 72).

#### Assessment of well-being

Every 4 hours during daytime wake periods, participants rated intensity of sleepiness, fatigue, and stress using computerized visual analogue scales (AsWin, programmed by Martin Rivers & Associates). The test battery required approximately 5 minutes per administration. Ratings were averaged across daytime periods (0700–2300 hours) for analysis.

### Statistics

The main analysis employed GLMM (SPSS 27) with Condition (ESD vs. control sleep), Study Day (representing the 8 intensive monitoring days), and Sex (female vs. male) as well as their interactions as fixed factors, and Participant ID as random factor. Baseline day was entered as a covariate to adjust for potential baseline differences between conditions. Satterthwaite’s approximation was used to calculate denominator degrees of freedom. Robust estimation was used to handle potential violations of model assumptions. To identify the best fitting model structure, 3 distributions in combination with or without log link function were tested (normal, gamma, and inverse Gaussian distribution in combination with identity or log link function) and homoscedasticity and normality were evaluated by plotting residuals against predicted values as well as histogram plots of residuals, respectively. A significant Condition × Study Day interaction effect was considered appropriate for follow-up with pairwise comparisons at single study days. A significant Condition × Sex effect was considered appropriate for follow-up with separate comparisons within females and males.

Data in the graphs are presented as estimated marginal means ± SEMs. The level of significance was set to an alpha value of rejection of *P* < 0.05.

## Supplementary Material

pgac004_supplemental_file

## Data Availability

All data are available in the manuscript and the supplementary material. Materials are available with appropriate material transfer agreements.

## Significance Statement:

Insomnia is a growing health problem worldwide and has emerged as a strong risk factor for the development of numerous diseases, including cardiometabolic, neurodegenerative, chronic pain, and autoimmune diseases. All of these diseases involve immunopathology, and almost all of them are characterized by pronounced sexual dimorphism. We, here, show that experimentally inducing insomnia-like sleep disturbances over several nights in healthy individuals causes inflammatory dysregulation in a sex-dependent manner. In females, proinflammatory markers were reduced, while males presented an increase in inflammatory markers and a reduction in the anti-inflammatory hormone cortisol. The findings suggest that the inflammatory consequences of sleep disturbances are sex-specific, which may be one reason for the pronounced sexual dimorphism in the many diseases associated with insomnia symptoms.
