# Correction to: Differential effects of an experimental model of prolonged sleep disturbance on inflammation in healthy females and males

**DOI:** 10.1093/pnasnexus/pgad455

**Published:** 2024-01-30

This is a correction to: Luciana Besedovsky, Rammy Dang, Larissa C Engert, Michael R Goldstein, Jaime K Devine, Suzanne M Bertisch, Janet M Mullington, Norah Simpson, Monika Haack, Differential effects of an experimental model of prolonged sleep disturbance on inflammation in healthy females and males, *PNAS Nexus*, Volume 1, Issue 1, March 2022, pgac004, https://doi.org/10.1093/pnasnexus/pgac004

During a retroactive audit conducted by *PNAS Nexus*, it was discovered that this paper was missing a statement acknowledging compliance with the *PNAS Nexus* Human and Animal Participants and Clinical Trials policy:

This study was approved by the IRB at the Beth Israel Deaconess Medical Center and all participants went through the informed consent procedures and signed the ICF.

This error has been corrected in the original article.

