# Supplementary material for: Differential effects of an experimental model of prolonged sleep disturbance on inflammation in healthy females and males
Source: PNAS Nexus. 2022 Mar 10;1(1):pgac004. doi: 10.1093/pnasnexus/pgac004 (PMC9648610; doi:10.1093/pnasnexus/pgac004)
Supplement: pgac004_Supplemental_File [file pgac004_supplemental_file.pdf]

Supplementary Information for

Differential effects of an experimental model of prolonged sleep disturbance on inflammation in healthy females and males

Luciana Besedovsky, Rammy Dang, Larissa C. Engert, Michael R. Goldstein, Jaime K. Devine, Suzanne M. Bertisch, Janet M. Mullington, Norah Simpson, Monika Haack

Monika Haack
Email: mhaack@bidmc.harvard.edu

This PDF file includes:

Figures S1 to S2
Tables S1 to S2
Supplementary Methods

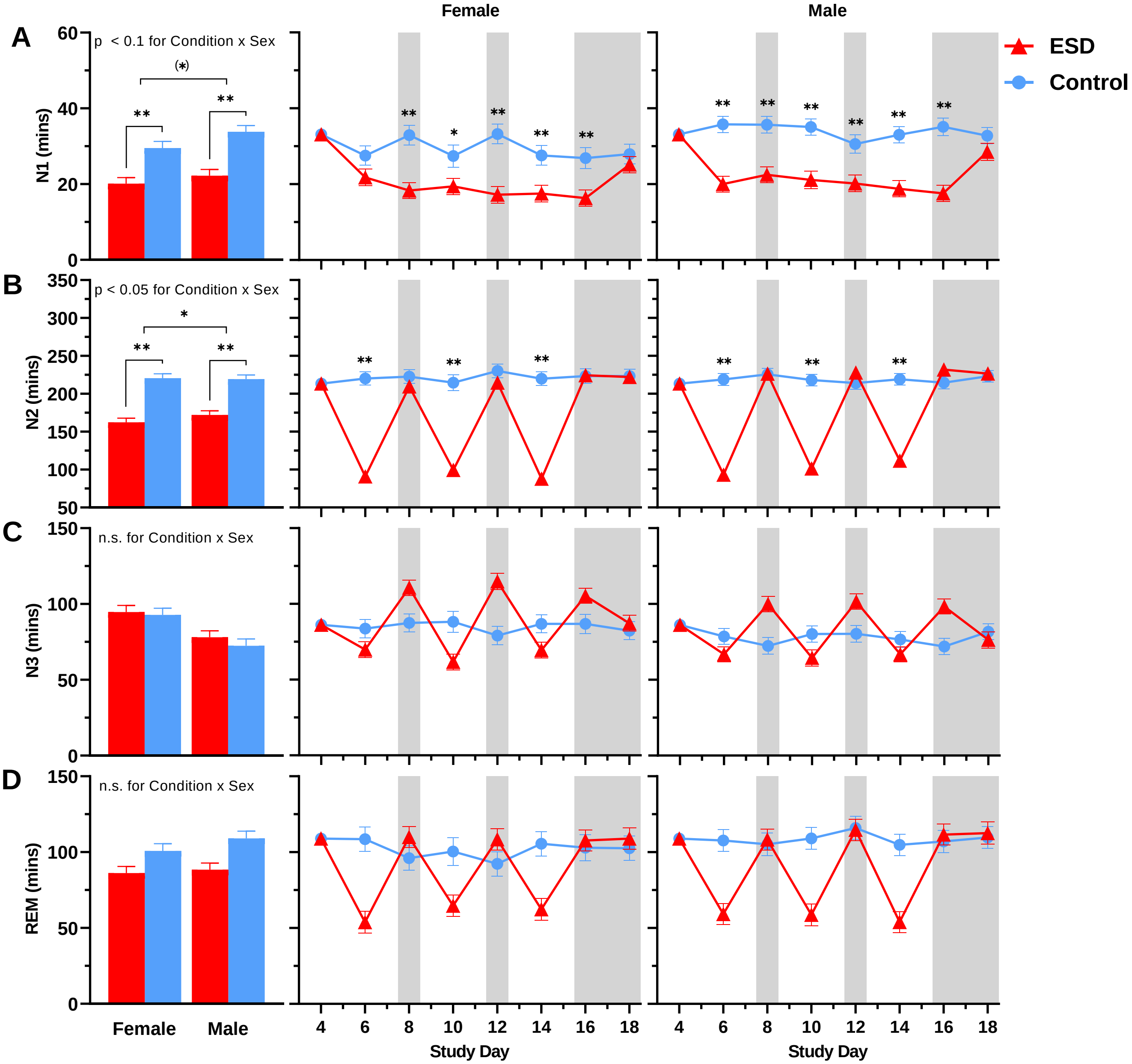

Figure S1. Sex differences in the effects of experimental sleep disturbance (ESD) on sleep architecture. Data present estimated marginal means ± SEM based on generalized linear mixed models for the time spent in (A) sleep stage N1, (B) sleep stage N2, (C) sleep stage N3, and (D) rapid-eye movement (REM) sleep for the ESD condition (red lines) and the control condition (blue lines) separated by sex; **p < 0.01, *p < 0.05, $^{(*)}$p < 0.10; n.s., not significant. n = 22-24.

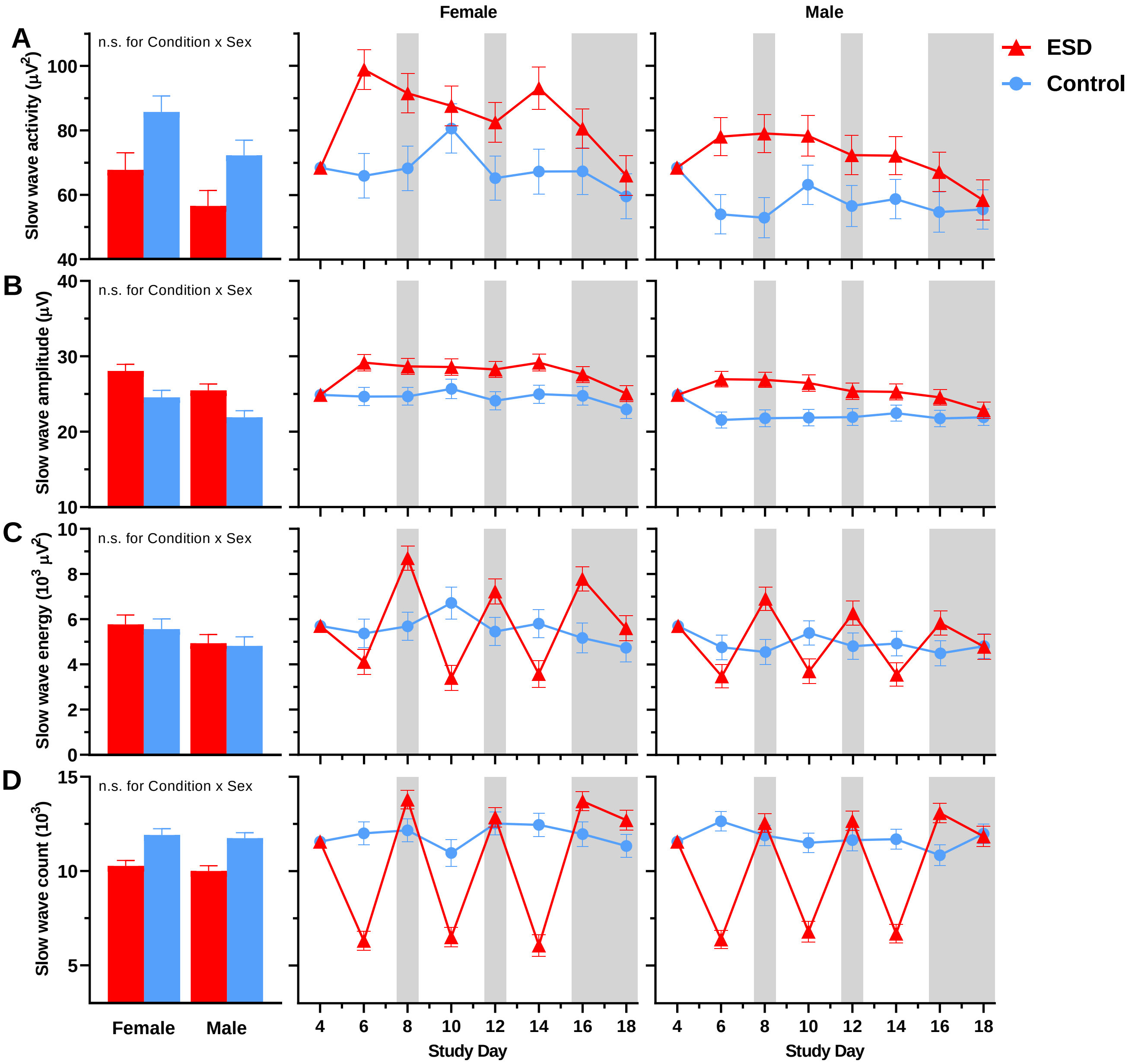

Figure S2. Sex differences in the effects of experimental sleep disturbance (ESD) on sleep depth. Data present estimated marginal means ± SEM based on generalized linear mixed models for (A) slow wave activity, (B) slow wave amplitude, (C) slow wave energy, and (D) slow wave count for the ESD condition (red lines) and the control condition (blue lines) separated by sex. n.s., not significant. n = 22-24.

Table S1. Participant characteristics.

		Control sleep	Sleep disturbance
N[A]		23	23
Sex	Female/Male	11/12	12/11
Age (yrs)	Mean ± SEM	28.4 ± 1.2	28.0 ± 1.2
BMI (kg/m^2)	Mean ± SEM	24.0 ± 0.8	23.8 ± 0.7
Menstrual cycle phase[B]	Follicular/Luteal	4/7	4/8
Hormonal contraceptive	N	2	2
Race	Black/White/Asian/Other[C]/	10/9/1/1/	10/10/1/1/
	Multiracial[D]/Unreported	1/1	1/0
Ethnicity	Hispanic/Non-Hispanic/Unreported	6/13/4	5/14/4

[A]Total N = 24 entering analysis. 22 participants completed both stays, 2 participants completed a single stay (1 sleep disturbance and 1 sleep control stay). [B]Menstrual cycle phase at day 1 of the experimental protocol. Two females did not have regular menstrual cycles; one naturally, one due to an intrauterine device. [C]Reported as Peruvian. [D]Reported as mixed Black and Alaska Native.

Table S2. Overview of the generalized linear mixed model (GLMM) analyses.

	Condition	Condition x Study Day	Condition x Sex	Distribution	Link function
**Sleep parameters**					
TST	$p < 0.001$	$p < 0.001$	n.s.	Normal	Identity
WASO	$p < 0.001$	$p < 0.001$	n.s.	Gamma	Log
N1 min	$p < 0.001$	$p = 0.005$	$p = 0.068$	Normal	Identity
N2 min	$p < 0.001$	$p < 0.001$	$p = 0.022$	Normal	Identity
N3 min	$p = 0.036$	$p < 0.001$	n.s.	Normal	Identity
REM min	$p < 0.001$	$p < 0.001$	n.s.	Normal	Identity
SWA	$p < 0.001$	$p = 0.002$	n.s.	Normal	Identity
Slow wave amplitude	$p < 0.001$	$p = 0.021$	n.s.	Normal	Identity
SWE	n.s.	$p < 0.001$	n.s.	Normal	Identity
Slow wave count	$p < 0.001$	$p < 0.001$	n.s.	Normal	Identity
**Humoral immune parameters**					
Unstimulated IL-6	$p = 0.005$	$p = 0.053$	$p < 0.001$	Gamma	Identity
Stimulated IL-6	n.s.	n.s.	$p = 0.007$	Normal	Identity
Plasma IL-6 levels	n.s.	$p = 0.004$	n.s.	Gamma	Identity
Plasma CRP levels	$p < 0.001$	$p = 0.006$	$p = 0.014$	Gamma	Identity
**Cellular immune parameters**					
WBC	n.s.	n.s.	$p = 0.048$	Gamma	Identity
Neutrophils	n.s.	n.s.	n.s.	Normal	Identity
Monocytes	$p = 0.062$	n.s.	$p = 0.057$	Gamma	Log
Lymphocytes	n.s.	n.s.	$p = 0.035$	Normal	Identity
$CD4^+$ T cells	n.s.	n.s.	$p = 0.035$	Normal	Identity
$CD8^+$ T cells	n.s.	n.s.	$p = 0.003$	Gamma	Log
**Cortisol levels and GC sensitivity**					
Cortisol levels 0700	n.s.	$p < 0.001$	$p = 0.005$	Normal	Log
Cortisol levels 1100	$p = 0.038$	$p < 0.001$	n.s.	Normal	Log
GC sensitivity	n.s.	n.s.	$p = 0.013$	Normal	Identity
**Subjective ratings**					
Sleepiness	$p < 0.001$	$p < 0.001$	$p < 0.001$	Normal	Identity
Fatigue	$p < 0.001$	$p < 0.001$	$p = 0.030$	Normal	Identity
Stress	$p = 0.026$	$p = 0.037$	n.s.	Normal	Identity

TST, total sleep time; WASO, wake after sleep onset; N1-N3, sleep stages 1-3, REM, rapid-eye-movement sleep; SWA, slow wave activity; SWE, slow wave energy; CRP, C-reactive protein; WBC, white blood cells; GC, glucocorticoid; n.s., not significant. The distribution and link function show the selected parameters for the GLMM analyses.

Supplementary Methods

Inclusion and exclusion criteria

Inclusion criteria were: age 18–45 years, body mass index (BMI) between 18.5 and 30 kg/m^2, daily sleep duration between 7 and 9 hours (verified by sleep diary data collected over seven days), beginning of the habitual sleep period within one hour of 2300 h (to ensure normal entrainment), blood chemistry levels within the normal range (including WBC and differential blood cell counts, T-cell subsets, thyroid hormones, glucose, insulin, creatinine, liver enzymes, erythrocyte sedimentation rate), and negative urine toxicology. Furthermore, female participants were eligible if they had regular menstrual cycles and no significant discomfort during pre-menses/menses. Exclusion criteria included presence or history of medical or psychiatric disorders (determined by diagnostic interviews, physician's medical history and physical examination), sleep disorders (based on questionnaires and polysomnography), pregnant/nursing status, regular medication use other than oral contraceptives, non-steroidal anti-inflammatory drug use in the two weeks prior to the study stays, and donation of blood or platelets three month prior to or in-between study stays. Blood tests and urine toxicology screening were repeated prior to the second 19-day in-hospital stay to ensure values remained in the normal range.

Research environment

Participants stayed in a private room at the Clinical Research Center at BIDMC throughout both in-hospital stays. Ambient room temperature was based on the individually tailored comfort level and kept stable throughout all study days. Participants were maintained on a balanced diet (NA^+ and K^+ controlled) and regimented fluid intake (no caffeine) in order to prevent changes in body weight/composition throughout the study. Meals and fluids were served at standardized times (0730 h breakfast, 1230 h lunch, 1830 h dinner, 2050 h snack).

To prevent sedentary conditions and maintain constant activity levels, the attending research assistant took participants to a 5 – 10 min walk within the Clinical Research Center or outside on hospital property every few hours during the day. Participants were encouraged to follow their pre-study exercise habits by visiting the hospital gym on the non-intensive monitoring days. During daytime periods, participants could have visitors and have access to email and phone, in order to minimize social isolation.
